# Coexistence of Autoimmune Encephalitis and Other Systemic Autoimmune Diseases

**DOI:** 10.3389/fneur.2019.01142

**Published:** 2019-10-31

**Authors:** Jing Zhao, Cancan Wang, Xiaolu Xu, Yuanxing Zhang, Haitao Ren, Zhixia Ren, Gai Li, Jiewen Zhang, Hongzhi Guan

**Affiliations:** ^1^Department of Neurology, People's Hospital of Zhengzhou University, Zhengzhou, China; ^2^Department of Neurology, People Hospital of Beijing Daxing District, Beijing, China; ^3^Department of Neurology, Peking Union Medical College Hospital, Chinese Academy of Medical Sciences, Beijing, China

**Keywords:** autoantibodies, encephalitis, autoimmune encephalitis, autoimmune diseases, comorbidities

## Abstract

**Background:** In recent years, the phenomenon of coexisting systemic autoimmune diseases (ADs) in patients with autoimmune encephalitis (AE) has been increasingly found, while its clinical significance remains unexplored. This study aimed to investigate the types and potential clinical associations of autoimmune comorbidities in patients with antibody-positive AE.

**Methods:** A retrospective cohort study of patients with antibody-positive AE was conducted from 2011 to 2018. The demographics, clinical characteristics, and follow-up data were reviewed.

**Results:** We enrolled 517 patients, among whom 45 were affected by one or more types of ADs, including Hashimoto's thyroiditis (HT) (*n* = 28), systemic lupus erythematosus (SLE) (*n* = 3), anaphylactoid purpura (*n* = 3), vitiligo (*n* = 3), Sjögren's syndrome (SS) (*n* = 2), chronic urticaria (*n* = 2), bullous pemphigoid (*n* = 1), uveitis (*n* = 1), myasthenia gravis (MG) (*n* = 1), and the coexistence of SLE and anaphylactoid purpura (*n* = 1). The proportion of patients with coexisting ADs was higher in those with anti–leucine-rich glioma-inactivated 1 (LGI1) encephalitis than in those with anti–N-methyl-d-aspartate receptor (NMDAR) encephalitis (13/111 vs. 16/307) (*P* = 0.021). In anti-NMDAR and anti-LGI1 encephalitis patients, there were no significant differences in the age at onset, sex ratio, proportion of patients with tumors, disease severity, or recurrence between the groups with and without ADs.

**Conclusions:** One or more types of ADs developed in AE patients, and patients with anti-LGI1 encephalitis had a higher frequency of autoimmune comorbidities than those with anti-NMDAR encephalitis. And we found that autoimmune comorbidities did not affect the clinical course of AE.

## Introduction

Since the discovery of anti–N-methyl-d-aspartate receptor (NMDAR) encephalitis in 2007, a series of types of autoimmune encephalitis (AE) mediated by autoantibodies against neuronal surface receptors and synaptic proteins have been discovered (1). The most common type of AE is anti-NMDAR encephalitis, followed by anti–leucine-rich glioma-inactivated 1 (LGI1) encephalitis and anti–gamma aminobutyric acid B receptor (GABA_B_R) encephalitis. Dubey et al. (2) reported that the incidence and prevalence of AE were comparable to infectious encephalitis. With the increase in the number of cases with confirmed AE, the phenomenon of the coexistence of AE and other systemic autoimmune diseases (ADs) has attracted scholars' attention, although its clinical significance remains unexplored. ADs have a complex, multifactorial basis, and both genetics and environmental factors contribute to the development of ADs. Specific autoantibodies and autoreactive T cells seem to be shared characteristics of AE and other ADs, while the potential pathogenesis of the coexistence remains to be further explored.

To the best of our knowledge, except for some case reports, there has been no study concentrating on autoimmune comorbidities in patients with AE. Our study recruited 517 antibody-positive AE patients, of whom 45 had autoimmune comorbidities. The main aim of this study was to investigate the possible clinical significance of the coexistence of AE and other ADs.

## Methods

### Patients

We eventually recruited 517 patients with AE who were admitted to Peking Union Medical College Hospital and the People's Hospital of Zhengzhou University from 2011 to 2018. All patients met the diagnostic criteria of definite AE (3), with positivity for autoantibodies and clinical manifestations and auxiliary examination results indicative of AE. Diagnoses of Hashimoto's thyroiditis (HT) were established by a combination of the presence of anti-thyroid antibodies (mainly antibodies to thyroid peroxidase and thyroglobulin), appearance on thyroid sonogram, and clinical features (4). Diagnoses of other ADs were based on the clinical manifestations, specific antibodies, biopsy results (if required), and reference to the diagnostic criteria of ADs by the corresponding specialists. This study was approved by the Ethics Review Committees of Peking Union Medical College Hospital and the People's Hospital of Zhengzhou University. All participants provided informed consent.

### Detection of Anti-neuronal Antibodies

All serum and cerebrospinal fluid (CSF) antibodies were measured using indirect immunofluorescence test (IIFT) kits purchased from EUROIMMUN AG (Lübeck, Germany) and used according to the manufacturer's instructions.

### Data Collection

Clinical data were collected from the recruited patients, including age, sex, the presence of coexisting ADs, clinical characteristics, tumors, the frequency of relapses, and the recurrence interval. Relapse was identified if there was a new symptom of AE or a worsening of preexisting symptoms after at least 2 months of stabilization or improvement.

### Statistical Analysis

The SPSS 21.0 statistical software package was used to perform statistical analysis. Continuous data are presented as the mean ± standard deviation, and categorical variables are presented as proportions and ratios. Independent sample *t*-test and chi-square test were used to compare the differences between the two groups as appropriate. A *P*-value (two-sided) < 0.05 was considered statistically significant.

## Results

### Clinical Characteristics and Types of AE of Recruited Patients

This study comprised 517 AE patients (249 females and 268 males). The clinical characteristics of the recruited patients are listed in Table 1. The types of AE consisted of anti-NMDAR encephalitis (*n* = 307), anti-LGI1 encephalitis (*n* = 111), anti-GABA_B_R encephalitis (*n* = 52), anti–contactin-associated protein-like 2 (CASPR2) encephalitis (*n* = 13), anti–α-amino-3-hydroxy-5-methyl-4-isoxazolepropionic acid 2-receptor (AMPA2-R) encephalitis (*n* = 6), anti–α-amino-3-hydroxy-5-methyl-4-isoxazolepropionic acid 1-receptor (AMPA1-R) encephalitis (*n* = 1), anti-IgLON5 encephalopathy (*n* = 3), anti–glutamic acid decarboxylase (GAD) encephalitis (*n* = 9), anti–myelin oligodendrocyte glycoprotein (MOG) antibody syndrome (*n* = 2), and AE with multiple autoantibodies, including the coexistence of anti-CASPR2 and anti-LGI1 antibodies (*n* = 7), anti-NMDAR and anti-GABA_B_R antibodies (*n* = 2), anti-NMDAR and anti-CASPR2 antibodies (*n* = 1), anti-NMDAR and anti-GAD antibodies (*n* = 1), anti-NMDAR and anti-aquaporin-4 (AQP4) antibodies (*n* = 1), and anti-LGI1 and anti-GAD antibodies (*n* = 1).

**Table 1:** Clinical characteristics of AE patients and comparison of the clinical characteristics between the groups with and without coexisting ADs in anti-NMDAR and anti-LGI1 encephalitis patients.

		**Age at onset (years)**	**Gender (women/men)**	**mRS**	**Proportion of tumors**	**Proportion of patients admitted into ICU**	**Proportion of mechanical ventilation**	**Proportion of relapse**	**Recurrence interval (months)**
Anti-NMDAR encephalitis (*n* = 307)	With ADs (*n* = 16)	25.13 ± 6.34	12/4	3.25 ± 1.61	1/16	5/16	4/16	3/11	8.00 ± 4.00
	Without ADs (*n* = 291)	26.41 ± 13.91	166/125	3.16 ± 1.52	40/291	83/291	58/291	67/269	9.36 ± 6.36
	*P*-value	0.479	0.157	0.815	0.631	1.000	0.864	1.000	0.663
Anti-LGI1 encephalitis (*n* = 111)	With ADs (*n* = 13)	55.54 ± 11.83	2/11	2.00 ± 0.91	0/13	1/13	0/13	3/11	7.67 ± 6.35
	Without ADs (*n* = 98)	59.00 ± 12.82	27/71	2.07 ± 1.11	4/98	5/98	5/98	26/70	11.50 ± 6.99
	*P*-value	0.358	0.547	0.824	1.000	0.535	1.000	0.767	0.374
Anti-GABA_B_R encephalitis (*n* = 52)		58.21 ± 9.96	16/36	3.00 ± 1.25	19/52	9/52	8/52	6/33	7.33 ± 3.01
Anti-CASPR2 encephalitis (*n* = 13)		50.00 ± 18.56	4/9	2.15 ± 1.07	1/13	0/13	0/13	3/10	8.00 ± 2.65
Anti–AMPA2-R encephalitis (*n* = 6)		58.50 ± 6.35	5/1	3.67 ± 0.82	3/6	2	2	2/4	7.50 ± 3.54
Anti–AMPA1-R encephalitis (*n* = 1)		58.00	0/1	3.00	0	0	0	–	–
Anti-IgLON5 encephalopathy (*n* = 3)		62.67 ± 1.53	1/2	2.67 ± 0.58	0	0	0	1/3	10
Anti-GAD encephalitis (*n* = 9)		45.22 ± 17.65	7/2	3.00 ± 1.41	0	2	0	0/3	–
Anti-MOG antibody syndrome (*n* = 2)		42.00 ± 4.24	0/2	2.00 ± 1.41	0	0	0	–	–

*AE, Autoimmune encephalitis; ADs, autoimmune diseases; NMRAR, N-methyl-d-aspartate receptor; LGI1, leucine-rich glioma-inactivated 1; mRS, modified Rankin Scale; ICU, intensive care unit; GABA_B_R, gamma aminobutyric acid B receptor; CASPR2, contactin-associated protein-like 2; AMPA2-R, α-amino-3-hydroxy-5-methyl-4-isoxazolepropionic acid 2-receptor; AMPA1-R, α-amino-3-hydroxy-5-methyl-4-isoxazolepropionic acid 1-receptor; GAD, glutamic acid decarboxylase; MOG, myelin oligodendrocyte glycoprotein*.

### The Types of ADs in Patients With Different Types of AE and the Percentages of Concomitant ADs in our Recruited Patients

Among the 307 anti-NMDAR encephalitis patients, 16 patients had ADs, including HT (*n* = 11), systemic lupus erythematosus (SLE) (*n* = 2), chronic urticaria (*n* = 2), and anaphylactoid purpura (*n* = 1). Among the 111 anti-LGI1 encephalitis patients, 13 patients had ADs, including HT (*n* = 6), vitiligo (*n* = 2), anaphylactoid purpura (*n* = 1), SLE (*n* = 1), the coexistence of SLE and anaphylactoid purpura (*n* = 1), Sjögren's syndrome (SS) (*n* = 1), and uveitis (*n* = 1). The proportion of patients with coexisting ADs was higher in those with anti-LGI1 encephalitis than in those with anti-NMDAR encephalitis (13/111 vs. 16/307) (*P* = 0.021).

Among the 52 anti-GABA_B_R encephalitis patients, 3 patients had HT, and 1 patient had SS. Among the 13 anti-CASPR2 encephalitis patients, 1 patient had HT, and 1 patient had bullous pemphigoid. Among the six anti–AMPA2-R encephalitis patients, one patient had myasthenia gravis (MG), and one patient had HT. Among the three anti-IgLON5 encephalopathy patients, one patient had vitiligo. Among the nine anti-GAD encephalitis patients, five patients had HT. Among the two patients with anti-MOG antibody syndrome, one patient had HT, and one patient had anaphylactoid purpura.

The percentages of concomitant ADs in our recruited patients and the background prevalence of some ADs in China are shown in Table 2 (5–11). The percentages of some concomitant ADs in our recruited patients are higher than the background prevalence in China.

**Table 2:** Types and percentages of concomitant autoimmune diseases.

**Type of autoimmune disease**	**Frequency**	**Percent**	**Background prevalence in China**	**References**
Hashimoto's thyroiditis	28	5.42%	1%	(5)
Systemic lupus erythematosus	4	0.77%	0.059%	(6)
Anaphylactoid purpura	4	0.77%	0.008–0.009%	(7)
Vitiligo	3	0.58%	0.56%	(8)
Sjögren's syndrome	2	0.39%	0.06–0.34%	(9)
Uveitis	1	0.19%	0.15%	(10)
Myasthenia gravis	1	0.19%	0.006%	(11)
Chronic urticaria	2	0.39%	–	–
Bullous pemphigoid	1	0.19%	–	–

Twenty-four patients had confirmed diagnoses of ADs before the onset of AE (the time interval between the onset of ADs and AE ranged from 2 months to 12 years), while 20 patients were diagnosed with AE and ADs simultaneously during hospitalization in our hospital; for 1 patient, the diagnosis of ADs was made 1 year after the onset of AE.

### Comparison of the Clinical Characteristics of Anti-NMDAR Encephalitis and Anti-LGI1 Encephalitis Patients Between the Groups With and Without Coexisting ADs

As shown in Table 1, there were no significant differences in the age at onset, sex ratio, proportion of patients with tumors, disease severity, proportion of patients who relapsed, or recurrence interval between the two groups.

## Discussion

This study aimed to explore the phenomenon of the coexistence of antibody-positive AE and other ADs. Previous studies have shown that one AD increases the chance of an additional AD (12), and we found that the percentages of some ADs in our recruited patients were higher than the background prevalence in China.

We also found that patients with anti-LGI1 encephalitis were more prone to having autoimmune comorbidities than patients with anti-NMDAR encephalitis (*P* = 0.021). Interestingly, previous studies showed that anti-LGI1 encephalitis was highly associated with several human leukocyte antigen (HLA) class II alleles, whereas anti-NMDAR encephalitis was not (13–15). Recently, Shu et al. (16) found that anti-NMDAR encephalitis was associated with the HLA class II allele DRB1^*^16:02, although the carrier frequency of this allele was rather low (≤ 30%). Compared with anti-NMDAR encephalitis, anti-LGI1 encephalitis seems to show a stronger genetic predisposition mediated by HLA class II alleles. In epidemiological and genetic studies, associations between HLA class II alleles and many ADs (such as HT and SLE) have been found (17, 18). Perhaps in anti-LGI1 encephalitis patients, the susceptibility genes played an important role in the formation of the autoimmune milieu, which was conducive to the coexistence of ADs.

In this study, the most frequent autoimmune comorbidity in patients with antibody-positive AE was HT (5.42%). We hypothesized that this finding may be related to the high prevalence of HT in the population (the prevalence of HT in our regions was approximately 1%). Tuzun et al. (19) showed that patients with anti-thyroid antibodies were inclined to develop AE. In encephalopathy, the presence of anti-thyroid antibodies is often taken as evidence of Hashimoto's encephalitis (HE). According to the diagnostic criteria for AE (3), when anti-thyroid and anti-neuronal antibodies exist simultaneously, antibody-positive AE should be diagnosed as the priority. Hence, before diagnosing HE, anti-neuronal antibodies should be detected to avoid misdiagnosis.

In this study, several AE patients had coexisting autoimmune skin or mucosal lesions, such as vitiligo, bullous pemphigoid, etc. Both the skin and the nervous system originate from the ectoderm, and some studies have found that the NMDA and LGI1 receptors can be expressed outside the nervous system (e.g., in human epidermal melanocytes and sebaceous glands in the skin) (20, 21). Skin lesions may lead to the exposure of autoantigens, which induce the production of autoantibodies in the case of the loss of immune tolerance.

AE can coexist with non-organ-specific ADs, such as SLE and SS. A previous study (22) showed that autoantibodies to the NMDAR NR1 subunit could be detected in patients with SLE, especially neuropsychiatric SLE (NPSLE). However, this detection method was ELISA instead of cell-based assay, and ELISA may have a risk of false positivity. Further studies are required to explore whether lupus antibodies could cross-react with the NMDAR NR1 subunit. As AE and NPSLE have similar clinical features, the detection of specific antibodies seems to be vital.

In this study, six patients with anti–AMPA2-R encephalitis were recruited, and three of them had thymoma. As a central lymphoid organ, the thymus provides an inductive environment for the development of T cells and induces the central tolerance (23). Thus, the presence of thymoma is often accompanied by ADs. One anti–AMPA2-R encephalitis patient with coexisting thymoma achieved clinical remission after thymectomy and immunosuppressive therapy. However, 1 year later, this patient manifested clinical symptoms of MG. Previous studies have shown that the mutilation of the autoimmune regulator (AIRE) gene was associated with thymoma-related MG (24, 25). Hence, that patient may have mutilation of the AIRE gene, which resulted in a disturbance of the homeostasis in the immune system.

In this study, there were no significant differences in disease severity or relapse between the two groups, indicating that the presence of ADs did not affect the progression or clinical outcomes of AE. Thus, the treatment strategies for AE patients are not affected by the presence of ADs.

## Conclusions

In summary, this was the first study to concentrate on the coexistence of AE and other ADs. Our study indicated that patients with anti-LGI1 encephalitis are more prone to having autoimmune comorbidities than patients with anti-NMDAR encephalitis. In addition, the presence of ADs does not affect the clinical course of AE.

## Data Availability Statement

The datasets generated for this study are available on request to the corresponding author.

## Ethics Statement

The studies involving human participants were reviewed and approved by Peking Union Medical College Hospital Ethics Review Committees and People's Hospital of Zhengzhou University Ethics Review Committees. Written informed consent to participate in this study was provided by the participants' legal guardian/next of kin.

## Author Contributions

HG and JZhang participated in the design of this study. JZhao, CW, XX, YZ, and HR collected clinical data. JZhao, CW, ZR, and GL analyzed the data. JZhao and CW draft the manuscript. All the authors read and approved the final manuscript.

### Conflict of Interest

The authors declare that the research was conducted in the absence of any commercial or financial relationships that could be construed as a potential conflict of interest.

## Abbreviations

ADs: autoimmune diseases
AE: autoimmune encephalitis
HT: Hashimoto's thyroiditis
SLE: systemic lupus erythematosus
SS: Sjögren's syndrome
MG: myasthenia gravis
NMRAR: N-methyl-d-aspartate receptor
LGI1: leucine-rich glioma-inactivated 1
GABABR: gamma aminobutyric acid B receptor
CSF: cerebrospinal fluid
IIFT: indirect immunofluorescence test
CASPR2: contactin-associated protein-like 2
AMPA2-R: α-amino-3-hydroxy-5- methyl-4-isoxazolepropionic acid 2-receptor
AMPA1-R: α-amino-3-hydroxy-5- methyl-4-isoxazolepropionic acid 1-receptor
GAD: glutamic acid decarboxylase
MOG: myelin oligodendrocyte glycoprotein
AQP4: aquaporin-4
HLA: human leukocyte antigen
HE: Hashimoto's encephalitis
NPSLE: neuropsychiatric systemic lupus erythematosus
AIRE: autoimmune regulator
mRS: modified Rankin Scale
ICU: intensive care unit.
